# Hidden intra-mandibular carcinoma cuniculatum appearing in a patient with metastatic prostate cancer: a case report

**DOI:** 10.1186/s12903-019-0745-1

**Published:** 2019-04-05

**Authors:** Elyette Broly, Philippe Barthélémy, Saïd Ciftci, Christian Borel, Martin Broly, Catherine-Isabelle Gros, Luc Marcellin, Fabien Bornert

**Affiliations:** 1 Department of Oral Medicine and Oral Surgery, University Hospital of Strasbourg, Strasbourg, France; 2 Faculty of Dental Surgery, University of Strasbourg, Strasbourg, France; 3 Department of Oncology, University Hospital of Strasbourg, Strasbourg, France; 4 University of Strasbourg, Faculty of Medicine, Strasbourg, France; 5 Department of Otorhinolaryngology, University Hospital of Strasbourg, Strasbourg, France; 6 Department of Medical Oncology, Paul Strauss Center, Strasbourg, France; 7 Department of Medical Biology, University Hospital of Nantes, Nantes, France; 8 Department of Oral Radiology, University Hospital of Strasbourg, Strasbourg, France; 9 INSERM (French National Institute of Health and Medical Research), “Regenerative Nanomedicine” Lab FMTS, UMR, 1260 Strasbourg, France; 10 Department of Pathology, University Hospital of Strasbourg, Strasbourg, France

**Keywords:** Second primary cancer, Carcinoma cuniculatum, Prevention, Mandible

## Abstract

**Background:**

Whereas the incidence of cancers increases, overall survival of cancerous patients improves. Preventing the onset of second primary cancer is a new public health challenge and requires a special attention from organ specialists. We report a rare case of carcinoma cuniculatum in a context of metastatic prostate cancer. No case was previously described. Diagnosis delay of carcinoma cuniculatum is frequent and particularly in case of endophytic intra-osseous topography. The aim of this case report is to remind that persistent pain requires medical evaluation to rule out any possibility of second primary cancer.

**Case presentation:**

A 78-year-old patient followed for a metastatic prostate cancer had been describing neuralgic dental pain in the lower posterior left quadrant for several months. Healing delay of tooth #37 (second left mandibular molar) extraction socket in the painful region led to an intra-alveolar incisional biopsy, which showed a tumor widely invading the mandibular body. Radiologic, histopathologic and clinical elements finally concluded to an intra-osseous carcinoma cuniculatum. Duration of total treatment (oral biopsy to hemimandibulectomy) and follow up were about five months and one year respectively. Patient died before reconstruction.

**Conclusion:**

This case recalls that any persistent tooth pain affecting cancer patients requires a thorough review to exclude any secondary primary cancers or any metastasis of the oral cavity and more specifically in jawbones.

## Background

As the incidence of cancer increases, survival of cancer patients improves. In 2008, 3 million people were considered as having a history of cancer in France [1]. In this context, maintaining patients quality of life, preventing recurrence and preventing the occurrence of secondary primary cancers (SPC = a new cancer other than a local recurrence or metastasis appearing beyond 2 months) is a public health challenge [2]. Studies dedicated to identifying risk factors for SPC do not yet allow accurate prediction [3].

Carcinoma cuniculatum, a very well differentiated sub-type of epidermoid carcinoma, is a rare, polymorphous invasive tumor, with a low risk of metastases [4]. Malignant tumors involving the jawbones are most often due to direct extension of the disease either from the oral cavity or from the surrounding tissue. Metastatic tumors constitute about 1% of all the malignancies occurring in the jaws and mostly affect the mandibular region and can deposit from any primary tumor [5–7].

The detection of SPC requires careful attention from the oncology team and organ specialists.

### Case presentation

**Fig. 1:**
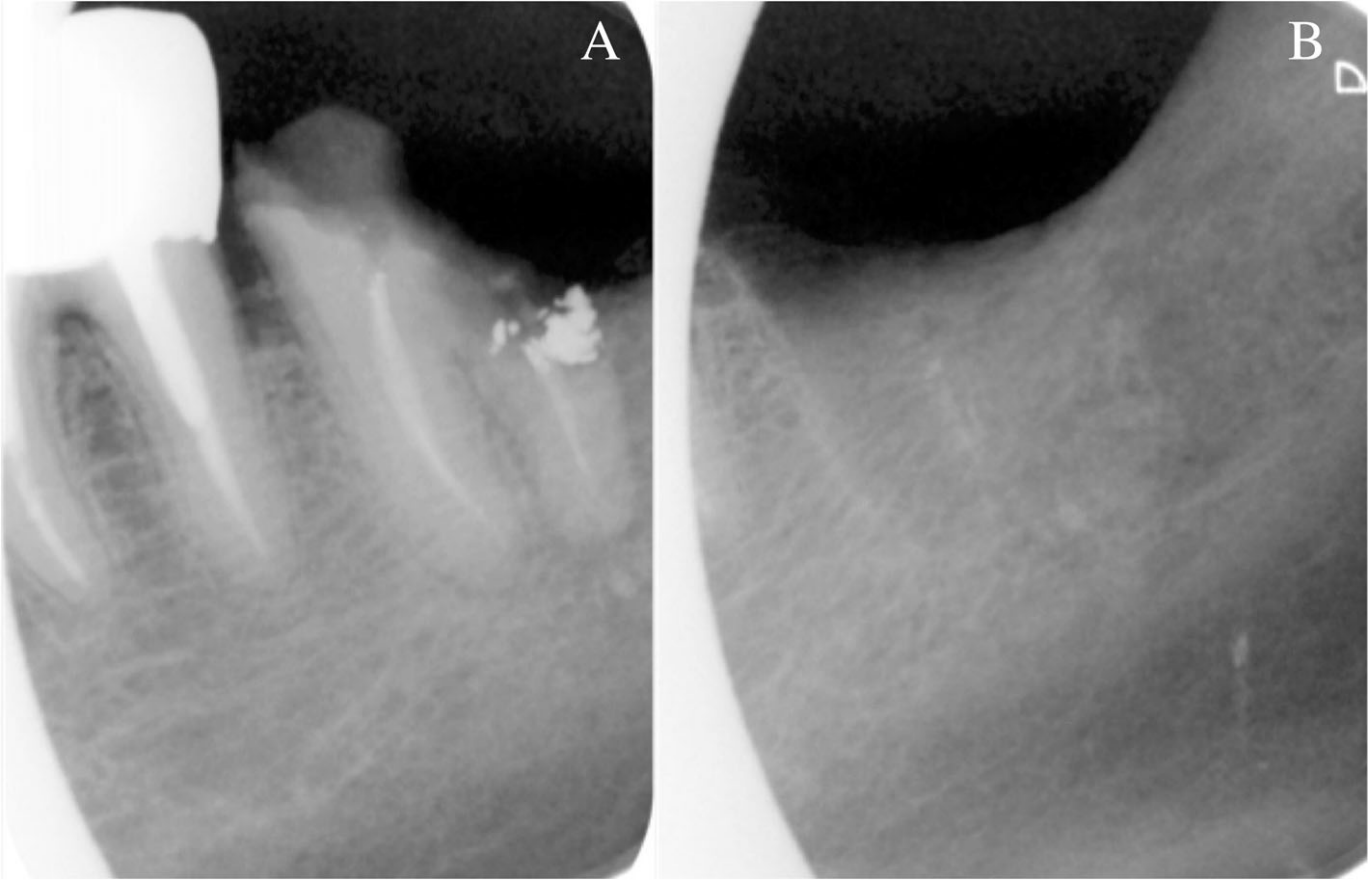
Retro-alveolar X-rays of tooth n°37. Retro-alveolar X-rays of tooth n°37: before (**a**) and one month after extraction (**b**). No sign of peri-apical radiolucency

A 78-year-old male patient followed for castration-resistant metastatic prostate cancer (disease progression despite androgen depletion therapy [8], also called CRPC) described highly debilitating and persisting neuralgic dental pain in the left posterior mandibular region. Patient’s oncological history was uncommon: one daughter, two brothers and two sisters died of various cancers. The patient smoked and consumed alcohol. Chemotherapy indicated for prostate cancer had been delayed due to the suspicion of odontogenic infection and the patient was referred to his dentist. After several unsuccessful antibiotic therapies, tooth #37 was finally removed (Fig. 1).

**Fig. 2:**
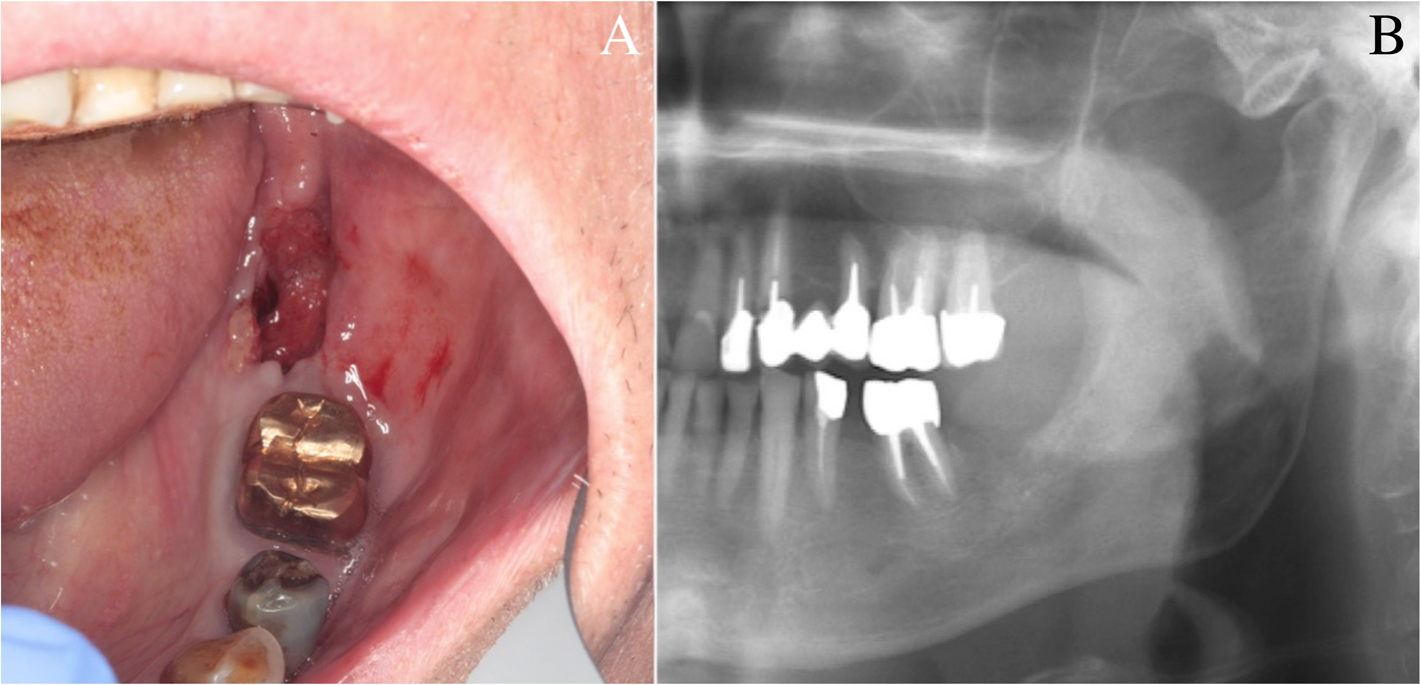
Intra-oral view and panoramic X-ray view of the patient. Intra-oral view (**a**) and panoramic X-ray (**b**), two months after extraction of tooth n°37. Healing delay of the extraction socket with slight bone exposure on the lingual side. Tongue and floor of the mouth were soft and unpainful

**Fig. 3:**
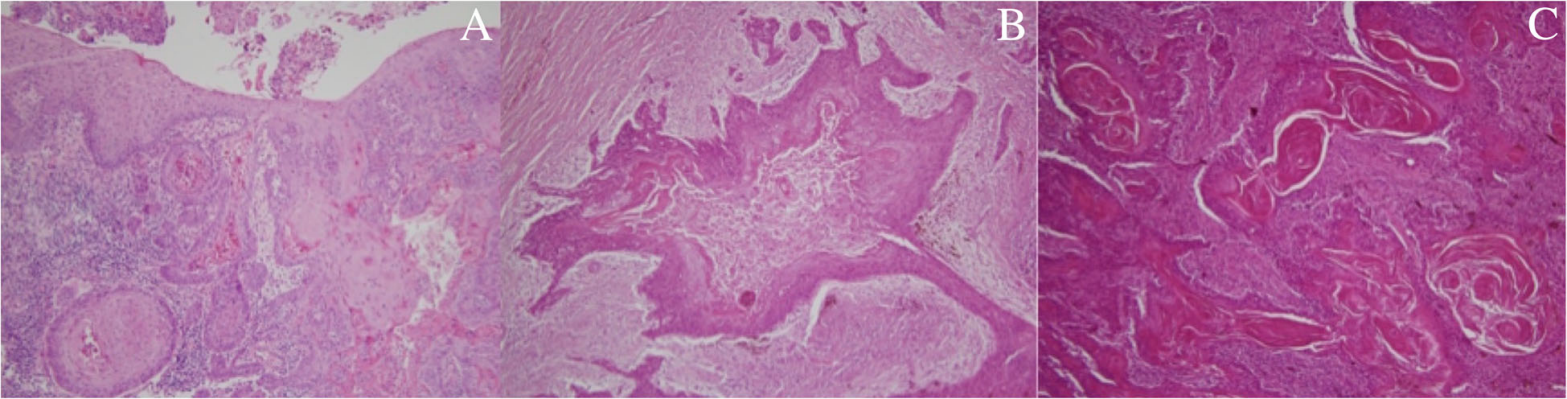
Microscopic views of biopsy (HE × 10), and tissue sample (HE × 40). Microscopic views of biopsy (HE X10) (**a**), and tissue sample (HE X40) (**b** and **c**). Papillomatosis with well differentiated squamous cells and infiltration of connective tissue below (**a**). Network of squamous carcinoma cells organized in sinuses, crypts and galleries filled with keratine (**b** et **c**)

**Fig. 4:**
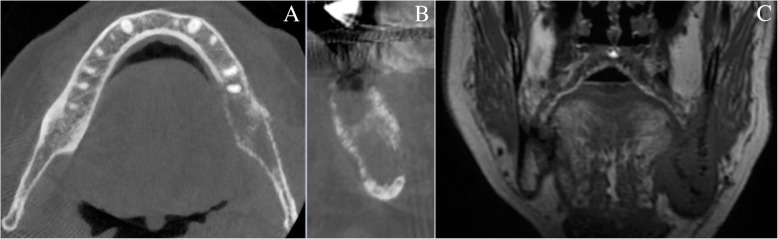
CBCT CBCT: horizontal slide of the mandible (**a**) and orthoradial reconstruction of left lower molar region (**b**). Osteolytic aspect of the jaw bone. Bis MRI. MRI (**c**): Coronal slide of the mandibular ramus in T1-weighted image. Tumoral invasion of whole trabecular bone and surrounding soft tissues (floor of the mouth and pterygomandibular raphe)

A 2-month healing delay of the extraction socket (Fig. 2) justified an alveolar incisional biopsy. A CRPC metastasis was suspected. Histopathology revealed a squamous cell carcinoma (SCC), classified cT4aN0M0, which largely invaded the left mandibular body according to imaging assessment (Figs. 3 and 4). Hemimandibulectomy and cervical dissection were performed to remove cancer and alleviate patient’s pain. Only one cervical node was infiltrated. The lesion was finally classified pT4N2M0. The patient refused radiotherapy. Given a nodal recurrence of the SCC in the cervical region, Paclitaxel-Carboplatin-Cetuximab chemotherapy in association to a second generation hormonal therapy for prostate cancer helped control the two cancerous diseases for about one year. Patient died before reconstruction. All radiological, anatomical and clinical elements retrospectively concluded to an intraosseous carcinoma cuniculatum (CC) (Fig. 3) [2]. X-rays performed during the dental follow-up did not allow to previously suspect any bone invasion of the CC (Figs. 1 and 2).

## Discussion and conclusion

CC, usually found on the sole of the foot, is a rare malignant tumor of the oral cavity (OC) classically not associated with tobacco nor alcohol [9]. As far as we know, only 61 cases of CC of the head and neck have been reported in the literature since 1977 [10–13]. Its diagnosis is difficult because only very few cell atypia can be found. Delayed diagnosis is common, especially in the case of intraosseous topography [14]; for this patient, the delay was 4 months. The anatomoclinical criteria were precisely defined in 1954 [15]. Maximum incidence occurs between 45 and 70 years of age [9]. There is a strong male predominance in all series reported [4, 16]. The distribution seems ubiquitous and all ethnic groups are equally affected. In most cases reported in the literature, a significant delay (3 to 8 months) elapsed between tumor onset and diagnosis. Size of the CC varies from 1 to 15 cm in its largest dimension [17]. For clinicians, the diagnostic difficulty lies in the clinical polymorphism of CC; moreover, its appearance can vary according to its evolutionary stage [4].

Macroscopically, the appearance is that of a verrucous tumor, exo- and / or endophytic, composed of lobules and epithelial masses of greyish-white appearance at the cut, with clear boundaries. On a slice section, budding zones are made of tissue hollowed out of anastomosing galleries, characteristic of CC [18]. Histological examination shows a well-differentiated epidermoid proliferation, without nucleo-cytoplasmic atypia, with the presence of galleries (cuniculi) proliferating in depth even though the basement membrane is always respected; the appearance of small break-in areas marks the transformation into squamous cell carcinoma (SCC) [9]. Histologically, some CC have transitional characteristics shared with well-differentiated SCC: for example, the presence of deep invasive casings consisting of clearly atypical squamous cells and more intense mitotic activity [19]. An anatomo-clinical confrontation, deep biopsies and total examination of the operative tissue specimen are the only way to reach a diagnosis of certainty.

Bone metastases are found mainly in lung, prostate, kidney, breast and thyroid cancers. Although metastases in the oral cavity are rare (1%), a prostate cancer (PC) metastasis was also considered in our patient. It occurs more frequently in bones than in soft tissues. To conclude to a metastatic invasion, the primary tumor must be histologically checked and the same histological features must be found for the primary tumor as well as the metastasis to exclude the possibility of direct local swarming of the primary tumor [20].

Histologically, by its invasive in depth evolution, in muscle tissue and / or in bone, intraosseous CC can also strongly mimic a primary intraosseous carcinoma [19]. The gingival epithelium continuity between the socket’s tissue and the mandibular lesion below made it possible to exclude this hypothesis (Fig. 3).

CC is an attenuated malignancy tumor, characterized by a slowly progressive local evolution, invariably invading the surrounding soft tissues, and rarely the underlying bone (10%). The development of lymph node metastases away from initial treatment is rare. CC is therefore a tumor of good prognosis after adequate treatment. However, there is currently no standardized protocol therapy for CC. Regardless of its location, exclusive surgical excision is the first line treatment in combination with close and prolonged monitoring [16–21]. Systematic lymph node dissection is in principle not required. However, it is advisable to propose neck surgical dissection in at least two cases: when there is palpable lymphadenopathy and when there is any doubt about the diagnosis of CC. Radiation therapy (RT) is contraindicated because it can induce a transformation of CC into SCC. However, RT may precede surgical excision in order to reduce the size of large CC; in very evolved cases it becomes the only therapeutic solution [22].

The etiopathogenesis of CC remains unclear. In fact, the lesion appears most often on healthy skin or mucosa, but many authors have published the occurrence of CC on pre-existing lesions, especially in areas exposed to trauma and inflammatory processes (15% of cases) [16]. CC of the oral cavity is not associated with usual carcinogenic factors (smoking, alcoholism, poor oral hygiene). Here the patient’s unique family history indicates a possible genetic predisposition but no more precise data were available in this regard. No case of CC seems to have been described in context of prostate cancer which is the most common malignancy in males [1, 14, 23]. Various studies carried out in the world didn’t show a global increased risk of SPC in the head and neck region in patients affected by prostate cancer [1]. However, some authors report a 0.2% SPC rate of neck and head SCC on a cohort of 19,406 cancer patients; prostate cancer was the most common primary cancer (27.5%). Out of 40 patients, all primary cancers combined, no CC was reported but the gingiva was affected in 11 cases [3, 23]. Moreover, the distribution of second cancer sites seems to be influenced by the distribution of primitive cancer sites [3]. Other studies are still necessary to better characterize SPC of the oral cavity.

This case recalls that any persistent tooth pain affecting cancer patients requires a thorough review to exclude any SPC or any metastasis of the oral cavity and more specifically in jawbones. Radiological semiology can be confusing at first sight. The medical and family history of the patient, as illustrated in this case, may probably constitute factors favoring the appearance of SPC. Knowledge of SPC thus must be fully integrated into the practice of oral surgeons. The detection of SPC of the oral cavity requires a good collaboration between medical oncologists and oro-dental practitioners. Future studies are still needed to determine more precisely the risk factors associated with oral SPC.

## Abbreviations

CC: Carcinoma cuniculatum
CRPC: Castration-resistant metastatic prostate cancer
OC: Oral cavity
PC: Prostate cancer
RT: Radiation therapy
SCC: Squamous cell carcinoma
SCP: Secondary primary cancer
